# LincRNA ZNF529-AS1 inhibits hepatocellular carcinoma via FBXO31 and predicts the prognosis of hepatocellular carcinoma patients

**DOI:** 10.1186/s12859-023-05189-0

**Published:** 2023-02-17

**Authors:** Yang Ma, Wan-liang Sun, Shuo Shuo Ma, Guanru Zhao, Zhong Liu, Zheng Lu, Dengyong Zhang

**Affiliations:** Department of General Surgery, The First Affiliated Hospital of Bengbu Medical College, Bengbu, 233000 Anhui China

**Keywords:** Hepatocellular carcinoma, Immune invasion, Tumour microenvironment, Prognosis

## Abstract

**Background:**

Invasion and metastasis of hepatocellular carcinoma (HCC) is still an important reason for poor prognosis. LincRNA ZNF529-AS1 is a recently identified tumour-associated molecule that is differentially expressed in a variety of tumours, but its role in HCC is still unclear. This study investigated the expression and function of ZNF529-AS1 in HCC and explored the prognostic significance of ZNF529-AS1 in HCC.

**Methods:**

Based on HCC information in TCGA and other databases, the relationship between the expression of ZNF529-AS1 and clinicopathological characteristics of HCC was analysed by the Wilcoxon signed-rank test and logistic regression. The relationship between ZNF529-AS1 and HCC prognosis was evaluated by Kaplan‒Meier and Cox regression analyses. The cellular function and signalling pathways involved in ZNF529-AS1 were analysed by GO and KEGG enrichment analysis. The relationship between ZNF529-AS1 and immunological signatures in the HCC tumour microenvironment was analysed by the ssGSEA algorithm and CIBERSORT algorithm. HCC cell invasion and migration were investigated by the Transwell assay. Gene and protein expression were detected by PCR and western blot analysis, respectively.

**Results:**

ZNF529-AS1 was differentially expressed in various types of tumours and was highly expressed in HCC. The expression of ZNF529-AS1 was closely correlated with the age, sex, T stage, M stage and pathological grade of HCC patients. Univariate and multivariate analyses showed that ZNF529-AS1 was significantly associated with poor prognosis of HCC patients and could be an independent prognostic indicator of HCC. Immunological analysis showed that the expression of ZNF529-AS1 was correlated with the abundance and immune function of various immune cells. Knockdown of ZNF529-AS1 in HCC cells inhibited cell invasion and migration and inhibited the expression of FBXO31.

**Conclusion:**

ZNF529-AS1 could be a new prognostic marker for HCC. FBXO31 may be the downstream target of ZNF529-AS1 in HCC.

**Supplementary Information:**

The online version contains supplementary material available at 10.1186/s12859-023-05189-0.

## Introduction

Hepatocellular carcinoma (HCC) is one of the most common malignant tumours in the world [1, 2]. The morbidity and mortality of HCC rank sixth and third, respectively [3]. The incidence of HCC in China is higher than that in Western countries [4, 5]. At present, the preferred method for the treatment of HCC is surgical resection for BCLC stage 0-B disease [6]. However, most patients have HCC metastasis at the time of treatment, and some patients have a high recurrence and metastasis rate after surgery, resulting in poor prognosis [7, 8]. In recent years, immunotherapy has been gradually applied by clinicians and has shown efficacy in the clinic [9], but some patients are not sensitive to immunotherapy. Therefore, it is necessary to identify key molecules related to the immune infiltrate of HCC, which provide better predictive targets for the prognosis and immunotherapy sensitivity of HCC.

ZNF529 is a member of the zinc finger protein (ZNF) family. The ZNF family plays a role through sequence-specific binding to downstream target molecules such as DNA, RNA, and DNA‒RNA, or binding to other zinc finger proteins (ZNFs) [10]. Many studies have shown that the abnormal expression of ZNFs plays an important role in the occurrence and development of HCC [11, 12]. The ZNF family also plays an important role in cancer prognosis, immune infiltration, the tumour microenvironment, epigenetics, and drug sensitivity [13]. ZNF529-AS1 (ZNF529-antisense 1) is the antisense strand of ZNF529 and plays the opposite role to ZNF529. We speculate that ZNF529-AS1 may play a regulatory role in the occurrence and development of HCC.

In this study, we first analysed the correlation of ZNF529-AS1 with clinicopathological characteristics, histological characteristics, survival prognosis, immune infiltration and sensitivity to chemotherapy in HCC patients in the database. In addition, we investigated the biological role of ZNF529-AS1 in regulating HCC cell invasion via FBXO31 in vitro.

## Materials and methods

### Patient datasets

We obtained RNA-seq data in TPM format from UCSC XENA (https://xenabrowser.net/datapages/), which were uniformly processed by the Toil program of TCGA and GTEx. The downloaded data were used to compare the expression level between 33 cancerous tissues and matched peri-cancerous tissues. We extracted the corresponding normal tissue data in LIHC and GTEx of TCGA, compared RNA-seq data in TPM format and performed logarithmic transformation on the expression between samples. We collected RNA-seq data and clinical information from a total of 424 HCC patients, including 50 paired peri-cancerous tissues for subsequent analysis. A total of 122 immunomodulatory factors, including MHC, receptors, chemokines, and immunostimulatory factors, were collected from the study by Charoentong et al. [14]. In addition, immune checkpoint molecules were retrieved from the study by Auslander et al. [15]. Spearman correlation analysis was used to explore the correlation between ZNF529-AS1 and the above factors in cancer.

### Survival analysis

The data of 374 HCC patients were collected and ranked according to the expression level of ZNF529-AS1 from low to high, with the median between the 187th and 188th patients. A value higher than this median was regarded as the high expression group; otherwise, it was regarded as the low expression group. The Kaplan‒Meier method [16] was used to analyse the relationship between ZNF529-AS1 expression and overall survival (OS), disease-specific survival (DSS) and progression-free interval (PFI) in HCC patients. The correlation between ZNF529-AS1 expression and DFS of HCC was analysed based on the GEPIA database (http://gepia.cancer-pku.cn/) [17]. Hazard ratios (HRs) and 95% confidence intervals were calculated using univariate survival analysis.

### Univariate and multivariate logistic regression analysis

We used univariate Cox regression analysis to calculate the relationship between the ZNF529-AS1 expression level and HCC prognosis. Multivariate analysis was used to evaluate whether ZNF529-AS1 was an independent prognostic factor for the survival of HCC patients. *p* < 0.05 was considered statistically significant.

### ZNF529-AS1-related functional enrichment analysis and drug sensitivity correlation analysis

Differentially expressed genes between the ZNF529-AS1 high expression and low expression groups were determined using the ‘limma’ R package [18]. The coexpression heatmap of ZNF529-AS1 and the top 20 differentially expressed genes was plotted according to Pearson correlation. Gene set enrichment analysis (GSEA) and GO and KEGG enrichment analyses were performed using the ‘clusterProfiler’ R package [19]. The differentially expressed genes between the ZNF529-AS1 high expression and low expression groups were analysed using the ‘DEseq2’ R package. All differentially expressed genes were pooled for GSEA, and the genes with |log2FC|> 1 and adjusted *p* value < 0.05 were selected for GO and KEGG enrichment analysis. The relationship between ZNF529-AS1 expression and drug sensitivity was analysed using the CellMiner database (http://discover.nci.nih.gov/cellminer/) [20, 21]. Data processing and plotting were performed through the “impute”, “limma” and “ggpubr" packages in R.

### Comprehensive analysis of the tumour microenvironment (TME) and immune cell infiltration

We obtained the gene set related to the cancer-immunity cycle from the website developed by Xu et al. (http://biocc.hrbmu.edu.cn/TIP/) [22]. We obtained the data of a set of gene signatures positively correlated with the clinical response to an anti-PD-L1 drug (atezolizumab) from the study by Mariathasan et al. [23]. GSVA was used to calculate the enrichment scores of gene signatures positively correlated with the cancer-immunity cycle and immunotherapy. We also collected gene sets related to immune function to analyse the activity of related pathways.

The stromal score, immune score and ESTIMATEScore of each HCC sample were estimated using the ESTIMATE algorithm (https://bioinformatics.mdanderson.org/estimate/). Single-sample gene set enrichment analysis (ssGSEA) was used to quantify the relative activity of various immune cells and immune functions in the TME, which was normalized to a unit distribution from 0 to 1. The Pearson correlation of ZNF529-AS1 with these immune cells and immune functions was analysed. A total of 22 inhibitory immune checkpoints with therapeutic potential were collected from the study by Auslander et al. [24], and their differences between the ZNF529-AS1 high expression and low expression groups were analysed.

### Cell culture and transfection

The HCC cells Hep3B and HUH7 and normal hepatocyte MHAs were obtained from the Shanghai Institutes for Biological Sciences, Chinese Academy of Sciences (Cat. No.: SCSP-5045, SCSP-526). The small interfering RNA (siRNA) of ZNF529-AS1 was purchased from Hippo Biotechnology Co., Ltd. Cells were transfected with siRNA as previously reported [25].

### Quantitative real-time PCR (qRT‒PCR)

The expression levels of ZNF529-AS and FBXO31 in HCC were determined by q-RT‒PCR according to protocols in a previous report [26]. The primers were as follows: ZNF529-AS1 forward 5′–3′: GACTCGCCAGGCTTAACTCA, reverse 5′–3′: GAGCTTCAGGGCTCTTTCGT; FBXO31, forward 5′–3′: TGGAGGACATCTTCCACGAG, reverse 5′–3′: GCATTGTCGTACTGACTGG; and GAPDH, forward 5′–3′: GGAGCGAGATCCCTCCAAAAT reverse 5′–3′: GGCTGTTGTCATACTTCTCATGG.

### Transwell assay

The invasion and migration assays of HCC cells were carried out 48 h after HCC cells were transfected with ZNF529-AS1. The details were the same as previously reported [27].

### Western blotting

Western blot analysis was performed using previously reported protocols [28]. The materials used included primary antibodies against FBXO31 (1:1000, Proteintech, USA) and GAPDH (1:500, Proteintech, USA) and a horseradish peroxidase (HRP)-linked secondary antibody (Cell Signalling, USA).

### Statistical analysis

All analyses were performed using the R programming language, and the data from different groups were compared by the Kruskal‒Wallis test, T test or Wilcoxon test. If not specified, *p* < 0.05 was considered statistically significant.

## Results

### Significant correlation of ZNF529-AS1 expression with immune factors in HCC

**Fig. 1:**
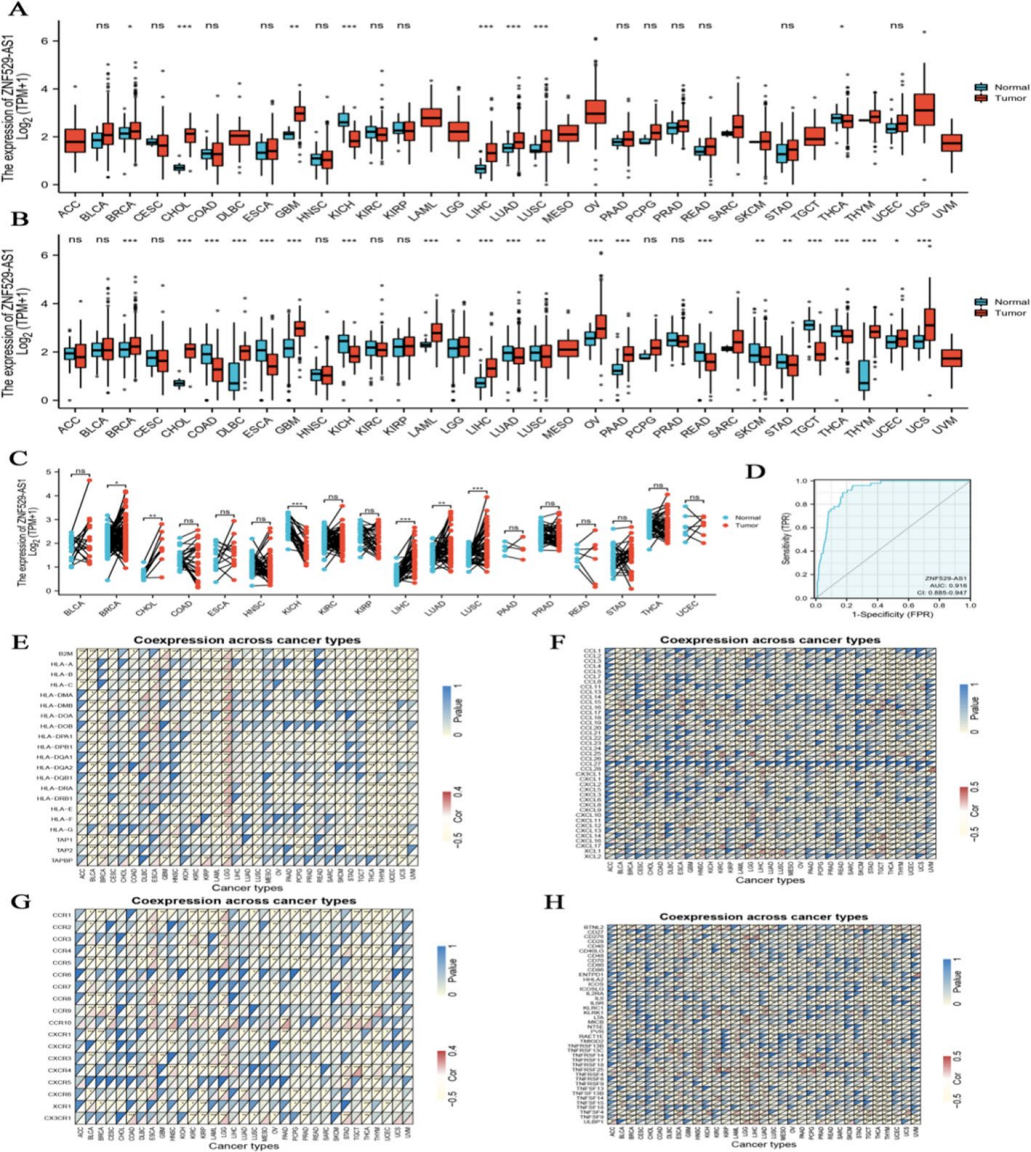
Expression and immunological characteristics of ZNF529-AS1 in different cancers. **A** The Wilcoxon rank sum test was used to analyse the differential expression of ZNF529-AS1 in tumour tissues and adjacent tissues. **B** The Wilcoxon rank sum test was used to analyse the differential expression of ZNF529-AS1 in normal adjacent tissues of GTEx-bound TCGA and tumour tissues of TCGA. **C** Differential expression of ZNF529-AS1 in tumour samples and matched adjacent samples. **D** ROC curve shows the efficiency of the ZNF529-AS1 expression level to distinguish HCC tissues from nontumor tissues. The X-axis denotes the false-positive rate, and the Y-axis denotes the true positive rate. **E**–**H** Heatmaps show the relationship between ZNF529-AS1 and **H** MHC molecules, **F** chemokines, **G** receptors and **H** immune checkpoints. For each relationship, the upper-left triangle is coloured to denote the p value, and the lower-right triangle is coloured to denote the correlation coefficient

Differential expression of ZNF529-AS1 was found in various tumour tissues and their peri-cancerous tissues (Fig. 1A, B), including breast cancer, HCC and lung adenocarcinoma. In HCC, ZNF529-AS1 expression was higher in cancerous tissues than in peri-cancerous tissues (Fig. 1C). The area under the curve (AUC) of ZNF529-AS1 was 0.916, indicating that ZNF529-AS1 may be a potential biomarker in HCC tissues (Fig. 1D). ZNF529-AS1 was significantly associated with MHC molecules, chemokines, receptors and immunostimulatory factors of cancer immune cells (Fig. 1E–H). These results show that the function of the tumour-associated molecule ZNF529-AS1 is associated with immune infiltration.

### Correlation analysis between ZNF529-AS1 expression and clinical characteristics of HCC

**Fig. 2:**
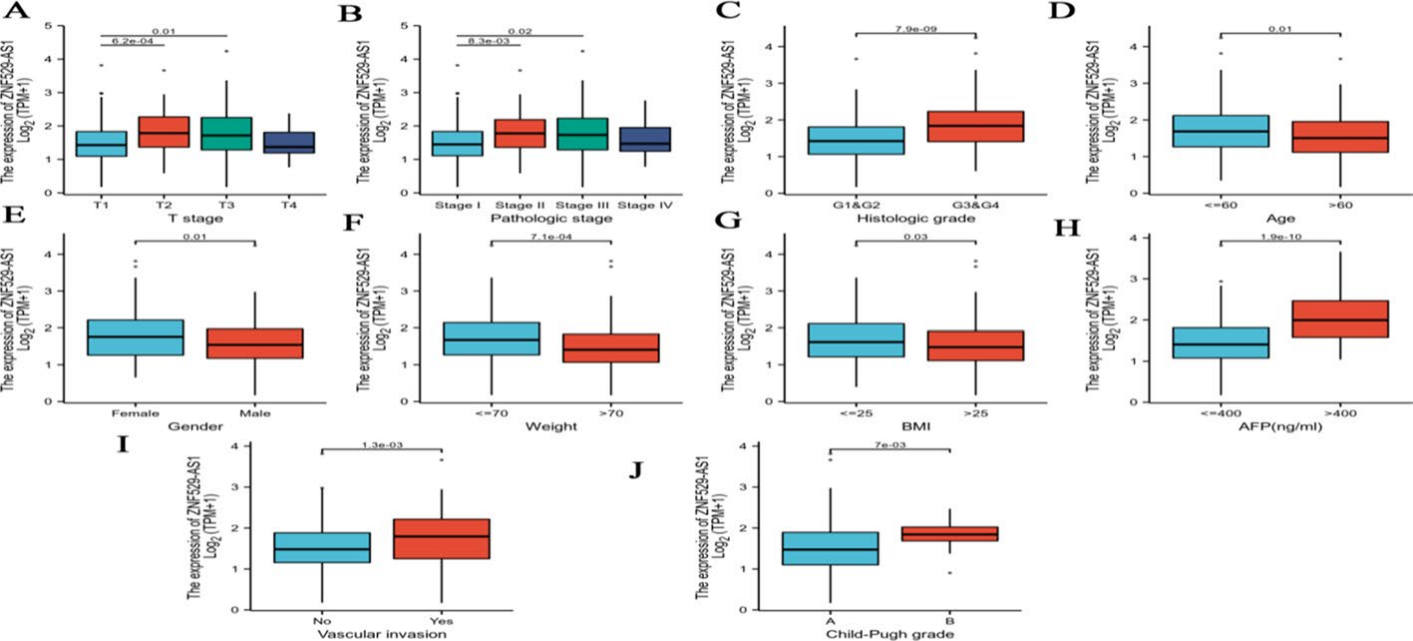
The relationship between ZNF529-AS1 expression and clinical characteristics of HCC patients: **A** T stage, **B** pathological stage, **C** histologic grade, **D** age, **E** sex, **F** weight, **G** BMI, **H** AFP, **I** vascular invasion, and **J** Child‒Pugh grade

The clinicopathological characteristics of HCC patients are shown in Table 1. The clinical and gene expression characteristics of 374 HCC patients were collected from the TCGA-LIHC database. According to the mean value of ZNF529-AS1 expression, HCC patients were divided into two groups: a high expression group (n = 187) and a low expression group (n = 187). The relationship between the ZNF529-AS1 expression level and the clinicopathological characteristics of HCC was analysed by the Wilcoxon rank sum test. By analysing the relationship between ZNF529-AS1 expression and clinical parameters, we found that ZNF529-AS1 expression was correlated with T stage (*p* < 0.05) (Fig. 2A), pathological stage (*p* < 0.05) (Fig. 2B), histological grade (*p* < 0.001) (Fig. 2C), age (*p* = 0.01) (Fig. 2D), sex (*p* = 0.01) (Fig. 2E), weight (*p* < 0.001) (Fig. 2F), BMI (*p* = 0.03) (Fig. 2G), AFP (*p* < 0.001) (Fig. 2H), vascular invasion (*p* < 0.01) (Fig. 2I), and Child‒Pugh grade (*p* < 0.01) (Fig. 2J) but not with other clinical characteristics (Additional file 1: Fig. S1). The relationship between the ZNF529-AS1 expression level and the clinicopathological characteristics of HCC patients was analysed by logistic regression (Table 2).

**Table 1:** Correlation between ZNF529-AS1 expression and clinicopathological characteristics in HCC

Characteristic	Low expression of ZNF529-AS1	High expression of ZNF529-AS1	*p*
n	187	187	
T stage, n (%)			< 0.001
T1	111 (29.9%)	72 (19.4%)	
T2	35 (9.4%)	60 (16.2%)	
T3	32 (8.6%)	48 (12.9%)	
T4	7 (1.9%)	6 (1.6%)	
N stage, n (%)			0.364
N0	130 (50.4%)	124 (48.1%)	
N1	1 (0.4%)	3 (1.2%)	
M stage, n (%)			1.000
M0	130 (47.8%)	138 (50.7%)	
M1	2 (0.7%)	2 (0.7%)	
Pathologic stage, n (%)			< 0.001
Stage I	104 (29.7%)	69 (19.7%)	
Stage II	33 (9.4%)	54 (15.4%)	
Stage III	34 (9.7%)	51 (14.6%)	
Stage IV	3 (0.9%)	2 (0.6%)	
Tumor status, n (%)			0.078
Tumor free	110 (31%)	92 (25.9%)	
With tumor	68 (19.2%)	85 (23.9%)	
Gender, n (%)			0.047
Female	51 (13.6%)	70 (18.7%)	
Male	136 (36.4%)	117 (31.3%)	
Race, n (%)			0.942
Asian	78 (21.5%)	82 (22.7%)	
Black or African American	9 (2.5%)	8 (2.2%)	
White	92 (25.4%)	93 (25.7%)	
Age, n (%)			0.055
≤ 60	79 (21.2%)	98 (26.3%)	
> 60	108 (29%)	88 (23.6%)	
Weight, n (%)			0.100
≤ 70	86 (24.9%)	98 (28.3%)	
> 70	91 (26.3%)	71 (20.5%)	
Height, n (%)			0.525
< 170	98 (28.7%)	103 (30.2%)	
≥ 170	74 (21.7%)	66 (19.4%)	
BMI, n (%)			0.347
≤ 25	85 (25.2%)	92 (27.3%)	
> 25	86 (25.5%)	74 (22%)	
Residual tumor, n (%)			1.000
R0	164 (47.5%)	163 (47.2%)	
R1	8 (2.3%)	9 (2.6%)	
R2	1 (0.3%)	0 (0%)	
Histologic grade, n (%)			< 0.001
G1	39 (10.6%)	16 (4.3%)	
G2	100 (27.1%)	78 (21.1%)	
G3	45 (12.2%)	79 (21.4%)	
G4	2 (0.5%)	10 (2.7%)	
Adjacent hepatic tissue inflammation, n (%)			0.049
None	62 (26.2%)	56 (23.6%)	
Mild	47 (19.8%)	54 (22.8%)	
Severe	14 (5.9%)	4 (1.7%)	
AFP (ng/ml), n (%)			< 0.001
≤ 400	129 (46.1%)	86 (30.7%)	
> 400	17 (6.1%)	48 (17.1%)	
Albumin (g/dl), n (%)			1.000
< 3.5	37 (12.3%)	32 (10.7%)	
≥ 3.5	123 (41%)	108 (36%)	
Prothrombin time, n (%)			0.455
≤ 4	112 (37.7%)	96 (32.3%)	
> 4	43 (14.5%)	46 (15.5%)	
Child–Pugh grade, n (%)			0.001
A	123 (51%)	96 (39.8%)	
B	4 (1.7%)	17 (7.1%)	
C	1 (0.4%)	0 (0%)	
Fibrosis ishak score, n (%)			0.551
0	38 (17.7%)	37 (17.2%)	
1/2	18 (8.4%)	13 (6%)	
3/4	12 (5.6%)	16 (7.4%)	
5/6	46 (21.4%)	35 (16.3%)	
Vascular invasion, n (%)			0.001
No	119 (37.4%)	89 (28%)	
Yes	41 (12.9%)	69 (21.7%)	
Age, median (IQR)	64 (55, 69)	59.5 (50, 67)	0.004

**Table 2:** ZNF529-AS1 expression associated with clinicopathologic characteristics (logistic regression)

Characteristics	Total (N)	Odds rati o(OR)	*p* value
T stage (T2 and T3 and T4 vs. T1)	371	2.375 (1.570–3.615)	< 0.001
N stage (N1 vs. N0)	258	3.145 (0.397–64.047)	0.324
M stage (M1 vs. M0)	272	0.942 (0.112–7.944)	0.953
Pathologic stage (Stage III and Stage IV vs. Stage I and Stage II)	350	1.595 (0.985–2.606)	0.059
Gender (Male vs. female)	374	0.627 (0.403–0.969)	0.036
Race (White vs. Asian and Black or African American)	362	0.977 (0.647–1.476)	0.913
Age (> 60 vs. ≤ 60)	373	0.657 (0.436–0.987)	0.044
Weight (> 70 vs. ≤ 70)	346	0.685 (0.447–1.046)	0.080
Height (≥ 170 vs. < 170)	341	0.849 (0.550–1.307)	0.456
BMI (> 25 vs. ≤ 25)	337	0.795 (0.517–1.220)	0.294
Residual tumor (R1 and R2 vs. R0)	345	1.006 (0.384–2.639)	0.990
Histologic grade (G3 and G4 vs. G1 and G2)	369	2.800 (1.812–4.371)	< 0.001
AFP (ng/ml) (> 400 vs.≤ 400)	280	4.235 (2.325–8.033)	< 0.001
Prothrombin time (> 4 vs. ≤ 4)	297	1.248 (0.759–2.056)	0.382
Albumin (g/dl) (≥ 3.5 vs. < 3.5)	300	1.015 (0.592–1.747)	0.956
Child–Pugh grade (B vs. A)	240	5.445 (1.941–19.396)	0.003
Vascular invasion (Yes vs. No)	318	2.250 (1.406–3.636)	< 0.001

### Correlation of high ZNF529-AS1 expression with poor prognosis of HCC

**Fig. 3:**
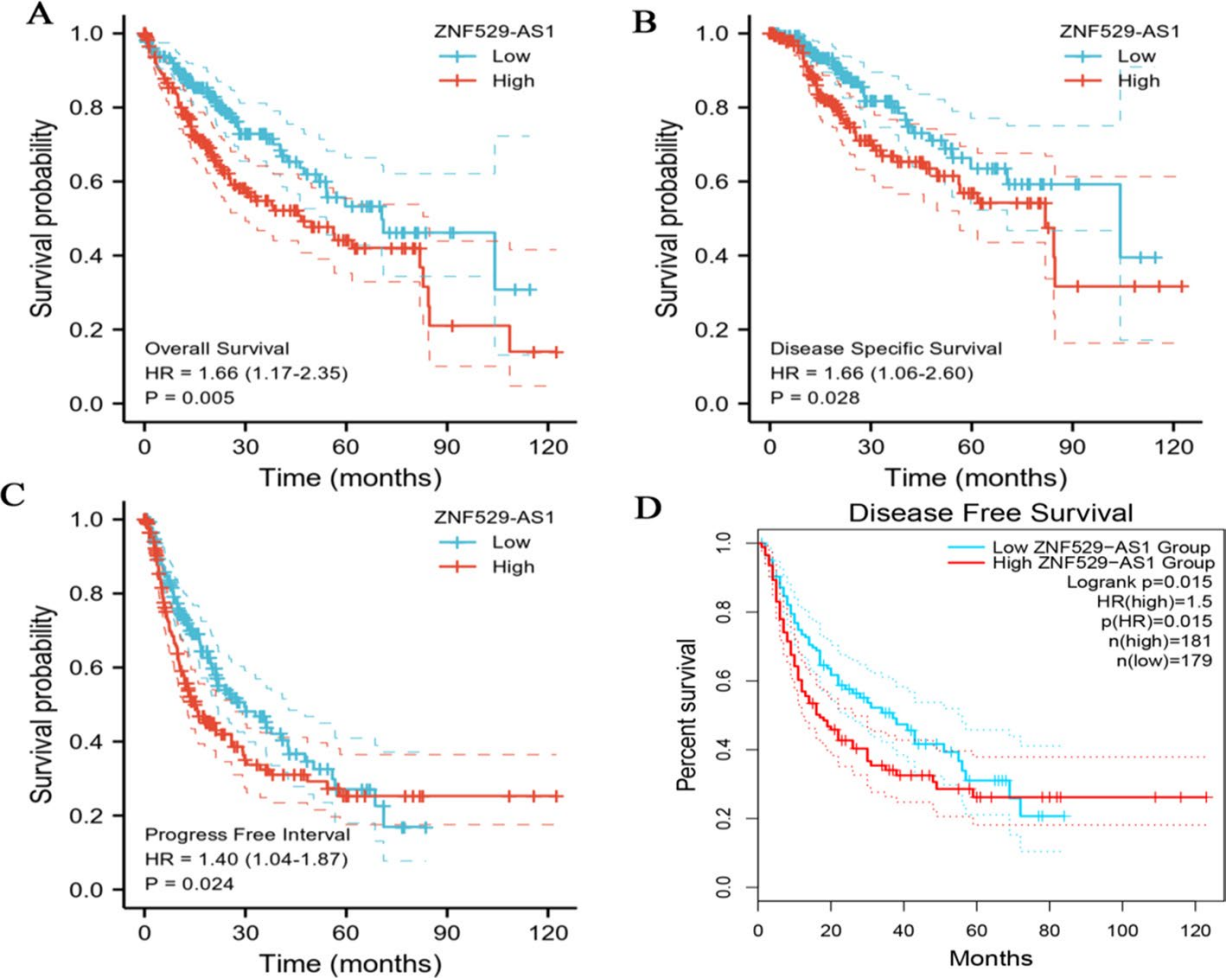
Kaplan‒Meier survival analysis of different ZNF529-AS1 expression states in LIHC from the TCGA dataset. **A** Overall survival; **B** disease-specific survival; **C** progression-free interval; **D** disease-free survival

Survival analysis of the TCGA-LIHC dataset showed that high ZNF529-AS1 expression in patients was significantly correlated with OS (*p* < 0.01) (Fig. 3A), DSS (*p* < 0.05) (Fig. 3B), and PFI (*p* < 0.05) (Fig. 3C) (poor prognosis). In addition, the analysis of the GIPIA2 database showed that high *ZNF529-AS1* expression in patients was significantly correlated with low DFS (*p* < 0.05) (Fig. 3D).

**Fig. 4:**
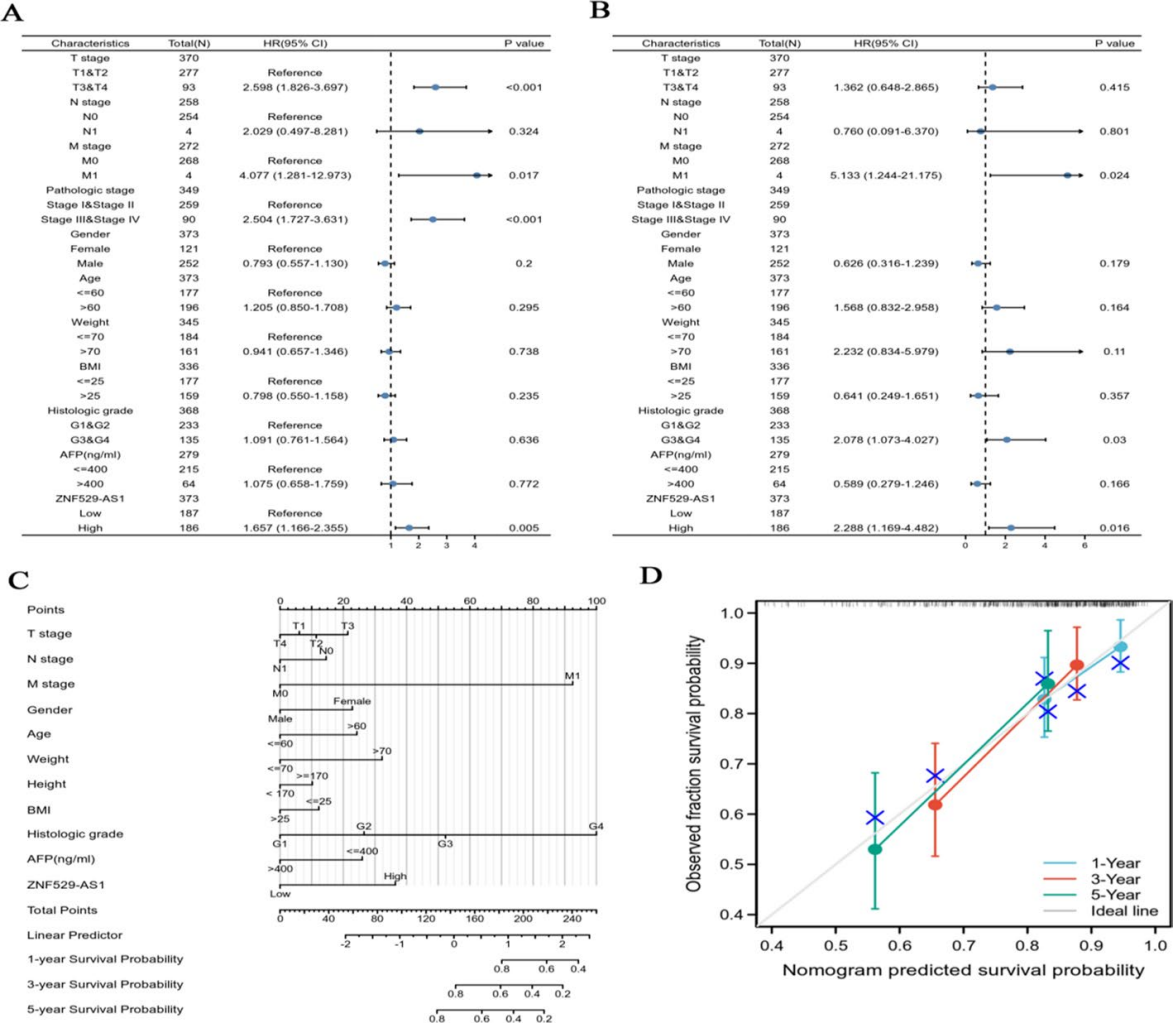
ZNF529-AS1 is an independent prognostic indicator of HCC and quantitatively predicts the survival probability of 1-, 3-, and 5-year OS in HCC patients. Univariate (**A**) and multivariate (**B**) COX regression analyses of ZNF529-AS1 and different clinical characteristics. **C** Nomograms predicting the 1-, 3-, and 5-year overall survival probabilities of HCC patients. **D** Calibration plot of the nomograms to predict the 1-, 3-, and 5-year overall survival probabilities of HCC patients

Univariate Cox analysis showed that T stage (*p* < 0.001), M stage (*p* < 0.05), pathological grade (*p* < 0.001) and ZNF529-AS1 expression (*p* < 0.01) were significantly correlated with OS in HCC patients (Fig. 4A). To further confirm the prognostic factors of HCC patients, multivariate risk analysis was performed by Cox regression, and the results showed that M stage (*p* < 0.05), histological grade (*p* < 0.05) and ZNF529-AS1 expression level (*p* = 0.013) were independent prognostic factors of OS (Fig. 4B). In addition, by constructing a Cox univariate regression model based on the DSS and PFI data of HCC patients and using Cox regression for multivariate risk analysis, we confirmed that ZNF529-AS1 expression was an independent prognostic factor of DSS (*p* < 0.05) and PFI (*p* < 0.01) in HCC patients (Additional file 1: Fig. S2). To further expand the clinical predictive value of ZNF529-AS1, we constructed a nomogram by combining multiple clinicopathological characteristics (Fig. 4C), and the nomograms of 1-, 3-, and 5-year survival probabilities were calibrated. The results showed that there was strong consistency between the prediction and the actual results (Fig. 4D).

### Negative correlation of ZNF529-AS1 with HCC chemosensitivity

**Fig. 5:**
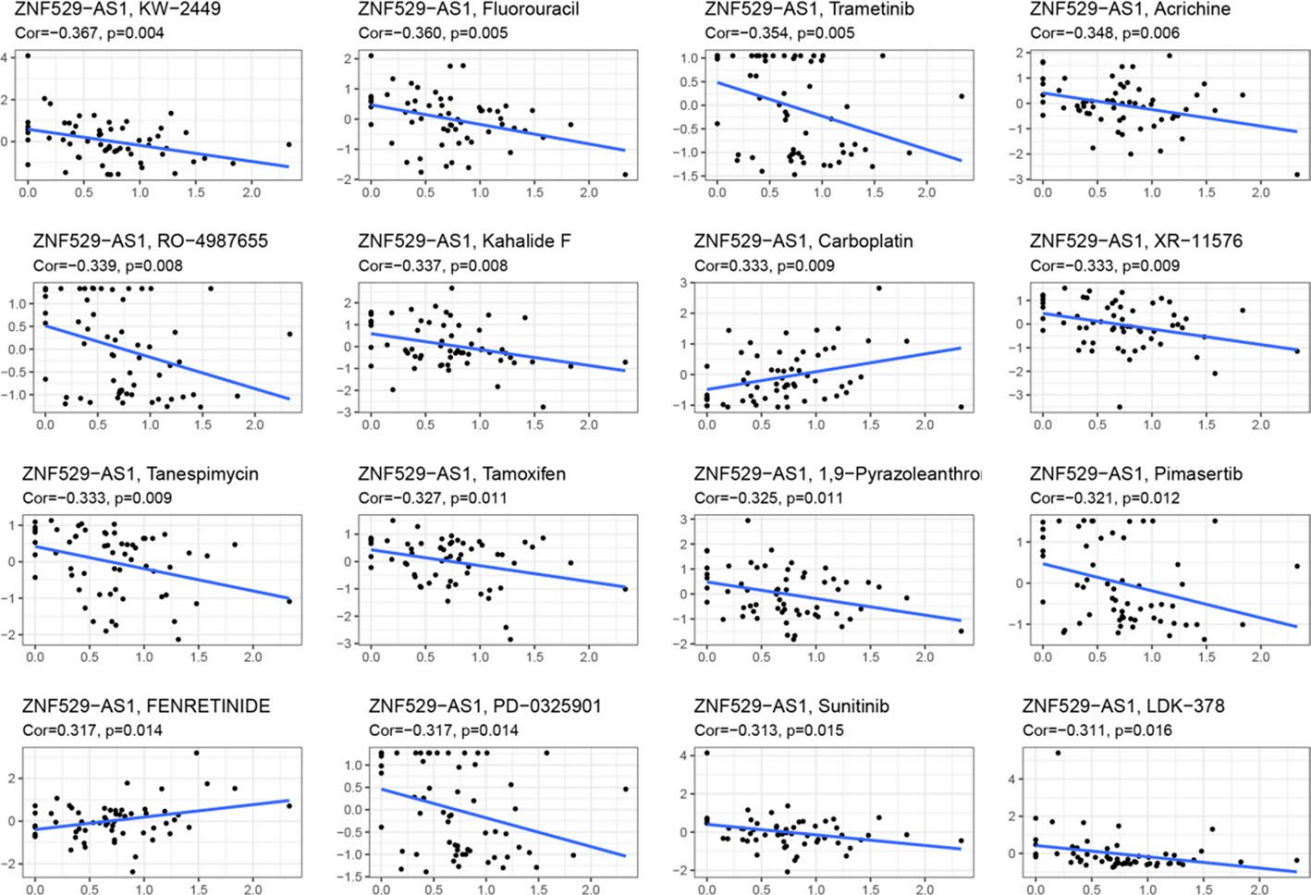
Correlation analysis between the ZNF529-AS1 expression level and drug sensitivity. The horizontal axis denotes gene expression, and the vertical axis denotes drug sensitivity

The correlation between ZNF529-AS1 and HCC chemosensitivity was explored through the CellMiner database. We identified 16 antitumour drugs that were significantly associated with ZNF529-AS1 expression. Among them, 14 drug sensitivities were significantly negatively correlated with ZNF529-AS1 expression, and 2 drug sensitivities were significantly positively correlated with ZNF529-AS1 (Fig. 5). The results showed that the expression level of ZNF529-AS1 was negatively correlated with sensitivity to most anticancer drugs (such as sunitinib, fluorouracil, and trametinib). Therefore, high expression of ZNF529-AS1 may affect chemotherapy efficacy against HCC.

### Functional enrichment of ZNF529-AS1-related genes in HCC

**Fig. 6:**
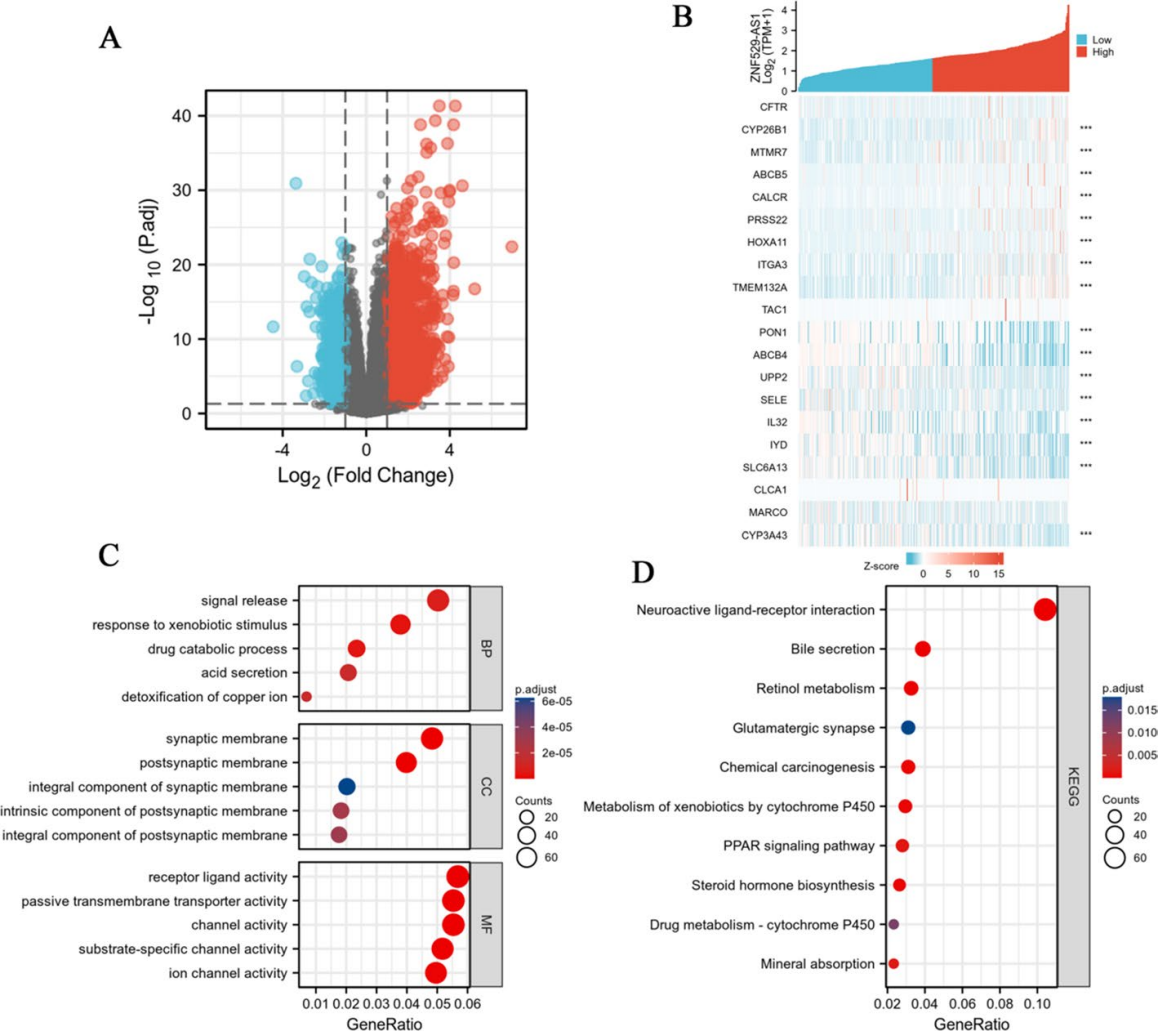
Functional enrichment analysis of ZNF529-AS1-related genes in HCC. **A** Heatmap of the differentially expressed genes, including 2455 upregulated genes and 579 downregulated genes; **B** heatmap of 20 differentially expressed genes, including 10 upregulated genes and 10 downregulated genes; **C** abundance of ZNF529-AS1-related genes associated with biological process; **D** KEGG pathway annotation

To elucidate the underlying molecular mechanism of the role of ZNF529-AS1 in HCC, the “DESeq2” R package was used to evaluate differentially expressed genes (DEGs) between the ZNF529-AS1 high expression and low expression groups. A total of 187 samples with high ZNF529-AS1 expression and 187 samples with low ZNF529-AS1 expression were analysed. A total of 3034 DEGs were identified, including 2,455 upregulated and 579 downregulated DEGs, which were statistically significant between the ZNF529-AS1 high expression and low expression groups (|log2FC|> 1, *p* < 0.05) (Fig. 6A). The heatmap showed the top 10 upregulated DEGs and the top 10 downregulated DEGs between the ZNF529-AS1 high expression and low expression groups (Fig. 6B). To predict the functional enrichment information between high expression and low expression of 4987 DEGs, the clusterProfiler package was used to perform GO and KEGG functional enrichment analysis (Additional file 2: Tables S1 and S2).

Thresholds of *p*. adj < 0.05 and FDR < 0.25 were used to screen significantly enriched items. Biological process (BP) association included signal release, response to xenobiotic stimulus, and drug catabolic process; cellular component (CC) included synaptic membrane, postsynaptic membrane, and integral component of synaptic membrane; molecular function (MF) included receptor ligand activity, passive transmembrane transporter activity, and channel activity (Fig. 6C). KEGG pathways included neuroactive ligand‒receptor interaction, bile secretion, and retinol metabolism (Fig. 6D).

### The relationship between ZNF529-AS1 expression and immune infiltration in HCC

**Fig. 7:**
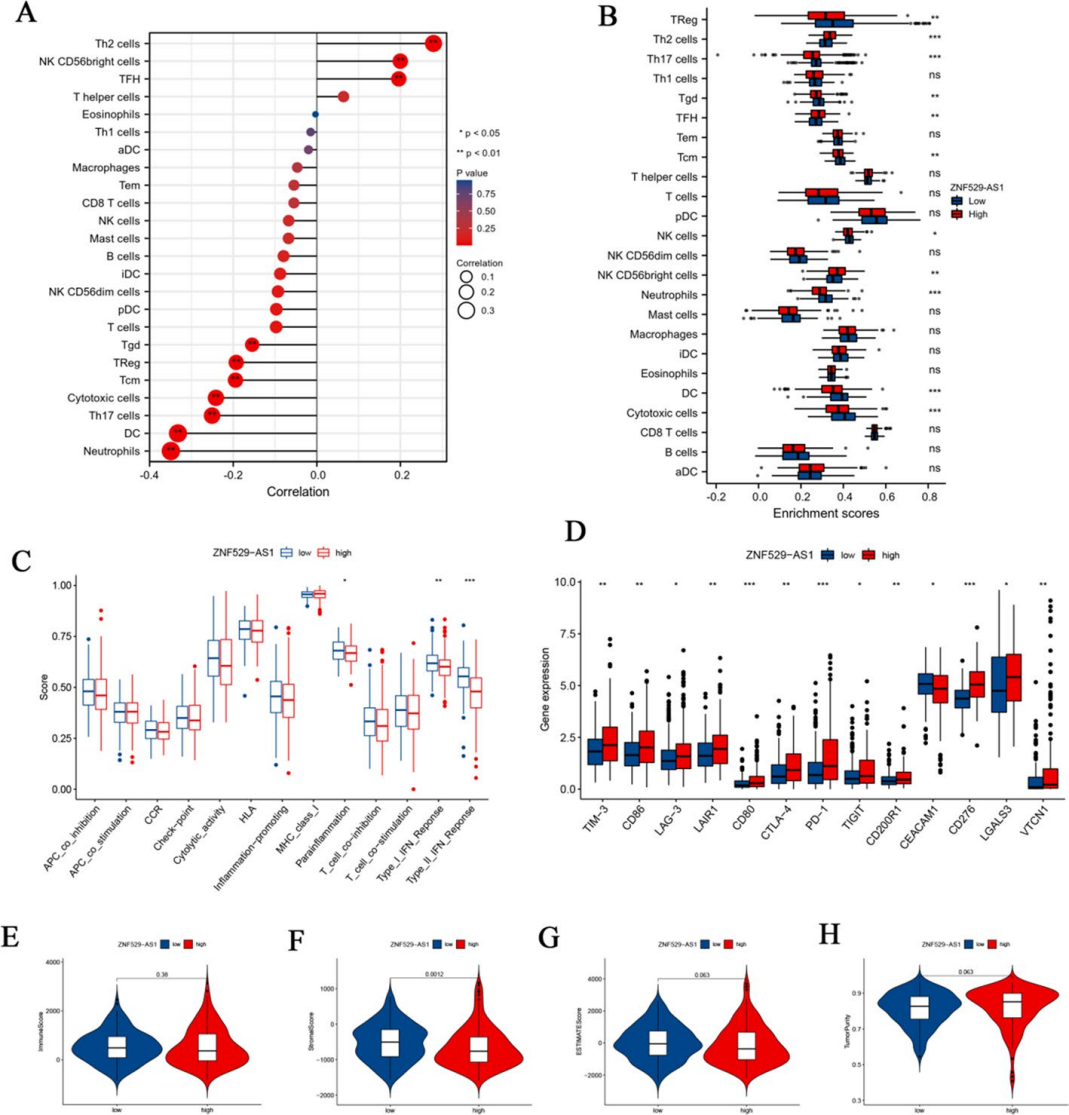
Immune infiltration analysis of ZNF529-AS1 in the HCC microenvironment. **A** Correlation analysis between ZNF529-AS1 and immune cells; **B** Wilcoxon test analysis of TIICs of HCC patients with high expression and low expression of ZNF529-AS1; **C** and **D** differential analysis of immune function and immune checkpoints between HCC patients with high expression and low expression of ZNF529-AS1; differences in the **E** immune score, **F** stromal score, **G** ESTIMATE score, and **H** tumour purity between the ZNF529-AS1 high expression and low expression groups (*p < 0.05; **p < 0.01; ***p < 0.001)

We systematically analysed the immune cells in the tumour and found that the expression of ZNF529-AS1 was positively correlated with the abundances of Th2 cells, NK CD56 bright cells and TFH cells and was negatively correlated with the abundances of Th17 cells, CD cells, and neutrophils (Fig. 7A). By comparing the proportions of immune cells in the *ZNF529-AS1* high-expression group and low-expression group in the TCGA dataset, we found that the levels of Th2 (*p* < 0.001), TFH (*p* < 0.01), NK CD56 bright cells (*p* < 0.01), and cytotoxic cells (*p* < 0.001) in the high ZNF529-AS1 expression group were higher, while the levels of Treg (*p* < 0.01), Th17 (*p* < 0.001), Tgd (*p* < 0.01), Tcm (*p* < 0.001), NK cells (*p* < 0.05), NK CD56 bright cells (*p* < 0.01) and DCs (*p* < 0.001) in the low ZNF529-AS1 expression group were higher (Fig. 7B). We further analysed the correlations between ZNF529-AS1 expression and immune cell markers, including B cells, CD8+ T cells, M1/M2 macrophages, tumour-associated macrophages (TAMs), neutrophils, natural killer cells and dendritic cells, in ccRCC using the GEPIA database. In addition, some different types of functional T cells, such as Th1, Th2, Th9, Th17, Th22, Tfh, exhausted T cells and Treg cells (Fig. 7C), were also detected. The expression of immune checkpoint proteins can be deregulated in tumours and is an important immune resistance mechanism. Therefore, we analysed the differences in immune checkpoint genes between the ZNF529-AS1 high expression and ZNF529-AS1 low expression groups. Compared with the low expression group, 11 immune checkpoint genes, including PD-1, CTAL4 and LAG-3, were significantly upregulated, and 2 immune checkpoint genes were significantly downregulated in the high expression group (Fig. 7D). Finally, the TME composition of HCC samples was analysed by ESTIMATE. The results showed that compared with the high ZNF529-AS1 expression group, the low ZNF529-AS1 expression group had a higher immune score (*p* = 0.38) (Fig. 7E), stromal score (*p* < 0.01) (Fig. 7F) and ESTIMATE score (*p* = 0.063) (Fig. 7G) and lower tumour purity (*p* = 0.063) (Fig. 7H), indicating that ZNF529-AS1 had a good ability to predict the TME components of HCC patients.

### Correlation analysis of ZNF529-AS1 in immunotherapy

**Fig. 8:**
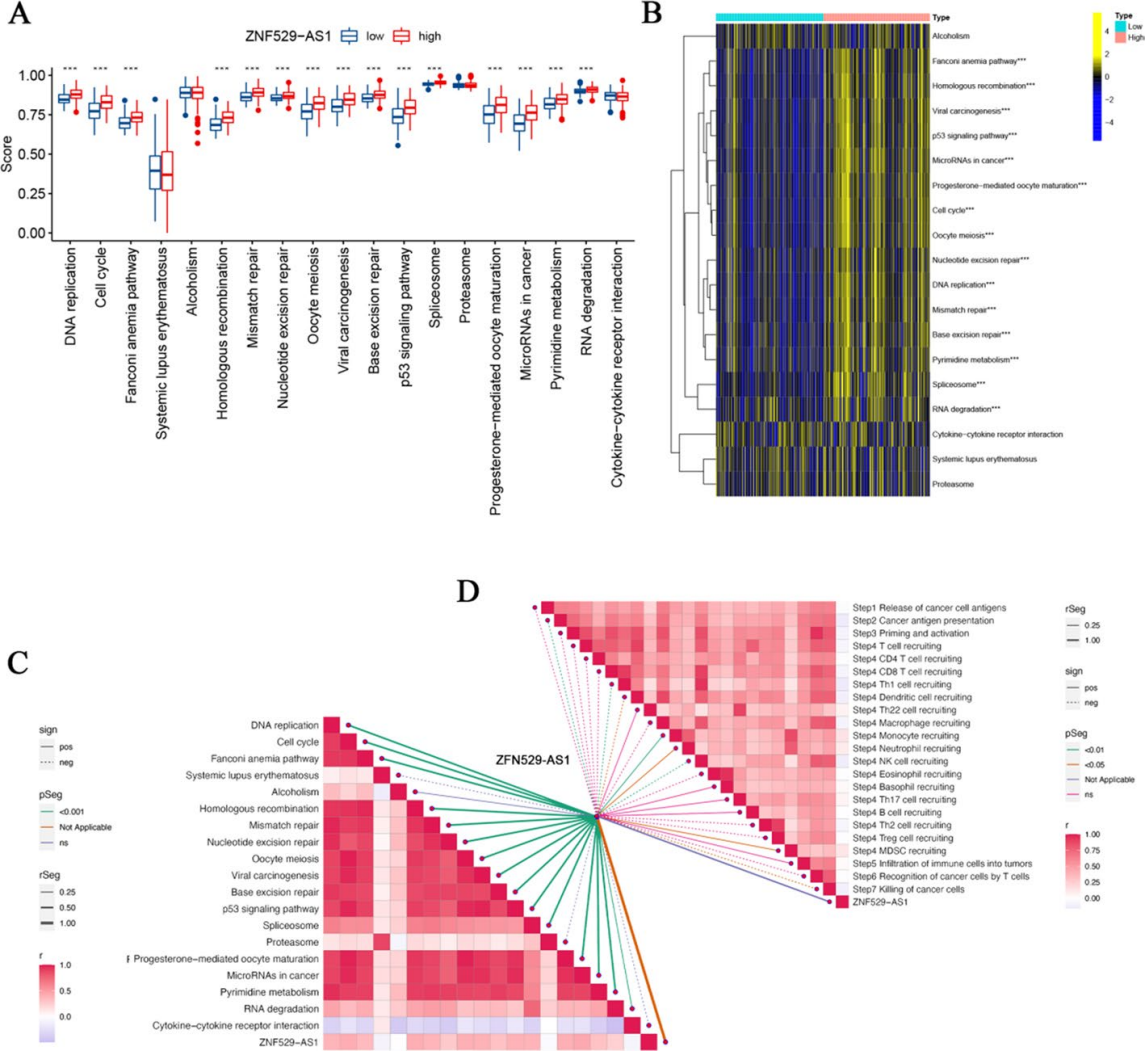
Evaluation of ZNF529-AS1 for predicting treatment response in HCC patients. **A** Difference analysis between ZNF529-AS1 and clinical response to HCC immunotherapy; **B** correlation between the high expression group and low expression group in the enrichment score of the immunotherapy prediction path; **C** correlation between ZNF529-AS1 and the enrichment score of the immunotherapy prediction pathways; **D** correlations between ZNF529-AS1 and various steps of the cancer-immunity cycle

Given the important role of immunotherapy in HCC, we analysed the correlation between the ZNF529-AS1 expression level and the pathway enrichment scores predicted by immunotherapy as well as key steps of the cancer-immunity cycle (Fig. 8C, D). The results showed that in the high ZNF529-AS1 expression group, risk scores were positively correlated with most immune pathways, including DNA replication, cell cycle, Fanconi anaemia pathway, homologous recombination, mismatch repair, nucleotide excision repair, oocyte meiosis, viral carcinogenesis, base excision repair, p53 signalling pathway, spliceosome, progesterone-mediated oocyte maturation, microRNAs in cancer, pyrimidine and RNA degradation (Fig. 8A, B). In addition, the high expression of ZNF529-AS1 was correlated with MDSC recruitment and monocyte recruitment at Step 4, a key step in the cancer-immunity cycle (Additional file 1: Fig. S3).

### ZNF529-AS1 regulated HCC cell invasion by targeting FBXO31

**Fig. 9:**
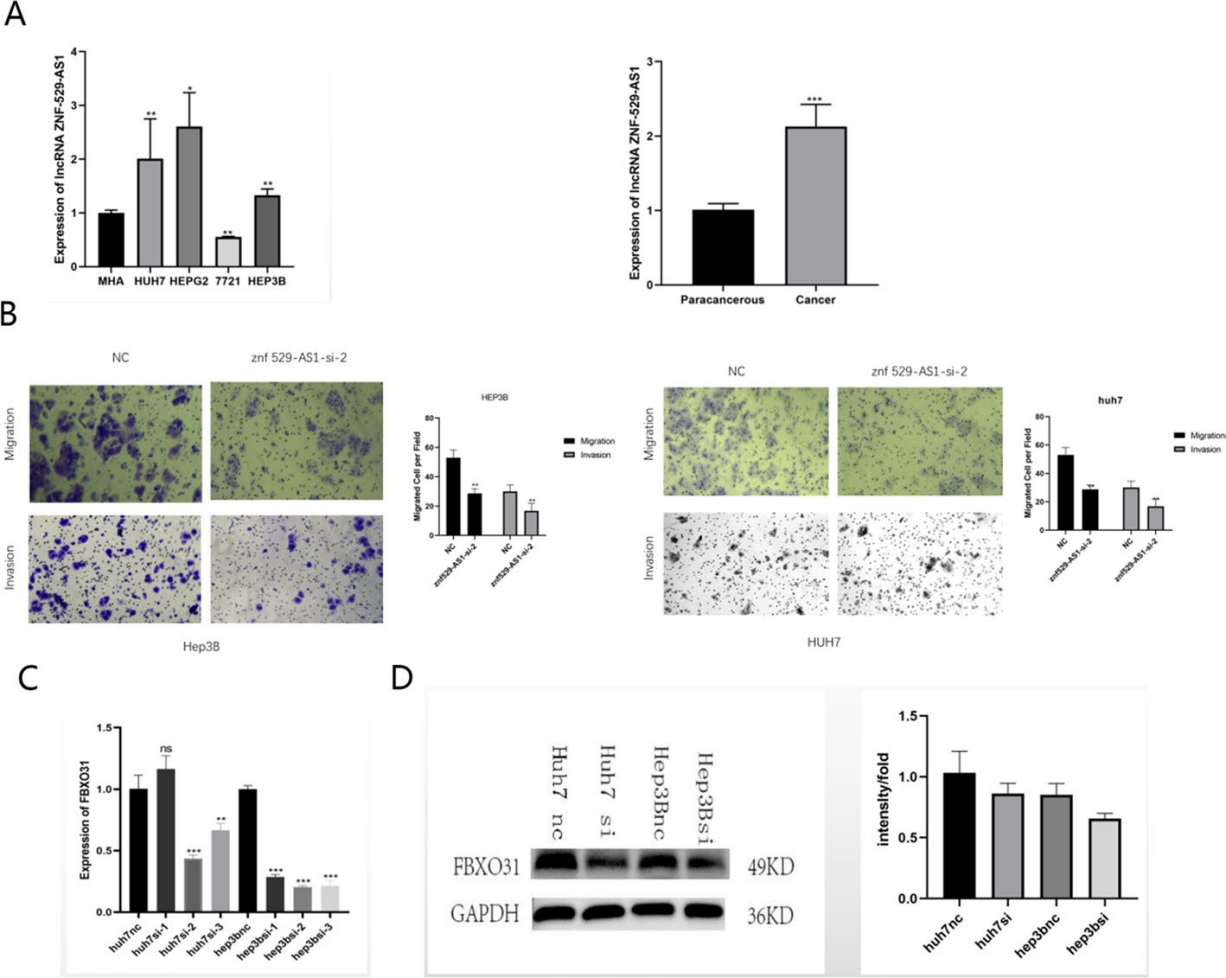
ZNF529-AS1 affects the invasion and migration of hepatocellular carcinoma. **A** Differential expression of ZNF529-AS1 in hepatocellular carcinoma cells and tissue was determined by qPCR assay. **B** The effect of ZNF529-AS1 on invasion and migration was determined by the Transwell assay. **C** and **D** Effects on downstream genes were determined by qPCR and WB assays after knocking down ZNF529-AS1. (*p < 0.05; **p < 0.01; ***p < 0.001)

The expression level of ZNF529-AS1 was relatively high in HCC tissues and HCC cells (Fig. 9A). Transwell assays showed that low ZNF529-AS1 expression inhibited the invasion and migration of tumour cells (Fig. 9B). After knockdown of ZNF529-AS1, the expression level of the downstream target gene FBXO31 decreased (Fig. 9C, D). It was inferred that FBXO31 may be a downstream target of ZNF529-AS1.

## Discussion

HCC is a common malignant tumour of the digestive tract and has an insidious onset and high postoperative recurrence and metastasis rates, leading to high mortality [29, 30]. As an important treatment method for cancer, immunotherapy has become the main force in the treatment of advanced HCC. However, many patients are not sensitive to immunotherapy, requiring us to identify potential prognostic and therapeutic targets.

In this study, we identified a novel tumour-associated molecule, ZNF529-AS1, in the TCGA database. ZNF529-AS1 is differentially expressed in various tumours, including HCC. At present, there are few studies on ZNF529-AS1. According to previous reports, ZNF family members play a regulatory role in tumorigenesis. For example, ZNF212 and ZNF367 can regulate the apoptosis of ovarian tumour cells and the proliferation of colorectal cancer cells, respectively [31, 32]. In the ZNF protein family, ZNF 471, ZNF1 and ZNF20 are involved in tumour progression through various pathways, including prognostic features, immune infiltration, TME, and tumour-related signalling pathways [33–35]. ZNF529-AS1 is the antisense strand of ZNF529, and its partial sequence is structurally opposite to that of the ZNF family, so it may have the opposite tumour regulation effect. We found that ZNF529-AS1 is upregulated in a variety of cancers in a TCGA database analysis. We analysed the role of ZNF529-AS1 in HCC and found that ZNF529-AS1 is closely correlated with T stage, M stage and pathological stage of HCC, indicating that ZNF529-AS1 may be potentially associated with the occurrence and development of HCC. By comparing the prognosis of HCC patients with high expression and low expression of ZNF529-AS1, we found a significant difference between the two groups (the higher the expression level was, the shorter the survival time), and ZNF529-AS1 was an independent factor affecting the prognosis of HCC patients. However, in our study, we used data from the TCGA and GTEx databases to analyse our results. Nonetheless, there are still some shortcomings, because there may be some asymmetry in the data, such as different TNM classification systems and different time courses, such as different TNM classification systems.

We speculate that ZNF529-AS1 may be a potential therapeutic target for HCC. By immunological correlation analysis, we found that ZNF529-AS1 is negatively correlated with immune-related molecules. ZNF529-AS1 inhibits the killing effect of immune cells on tumours and promotes the occurrence and development of HCC in vivo. Moreover, high expression of ZNF529-AS1 can affect the expression of CD80, PD-1 and CD276 in immune checkpoint cells, thereby promoting the occurrence of HCC. ZNF529-AS1 plays an important role in the immune system of HCC, not only affecting the cellular immune process in HCC but also affecting the immune checkpoint activity of HCC. ZNF529-AS1 promotes the occurrence and development of HCC through this dual role. At the same time, through targeted drug sensitivity analysis, we found that the expression of ZNF529-AS1 was negatively correlated with sensitivity to multiple targeted drugs, indicating that ZNF529-AS1 may be a potential therapeutic target for HCC. Knockdown of ZNF529-AS1 expression may improve the sensitivity to targeted drug therapy for HCC, promote the activation of the body’s immune system, inhibit the occurrence and development of HCC, enhance clinical efficacy, and improve patient prognosis. Additionally, this depends on tumour heterogeneity. Some patients were not sensitive to ZNF529-AS1.

The zinc lipoprotein family has an important role in the immune infiltration of tumours. In this study, we made a correlation prediction for ZNF529-AS1 in the immune infiltration of tumours, and the results showed that ZNF529-AS1 could affect the immune infiltration of hepatocellular carcinoma. Moreover, we correlated the functional expression of FBXO31 with ZNF529-AS1 by predicting that FBXO31, as a member of the forkhead box protein family, was reported to play an important role in immune infiltration of tumours[36], such as in hepatocellular carcinoma, lung cancer, breast cancer and colorectal cancer[37–40], in which forkhead box proteins play an important role in the function of immune infiltration. Second, we also investigated the function of ZNF529-AS1 at the cellular level. qRT‒PCR showed that ZNF529-AS1 was relatively highly expressed in HCC cells and tissues. Moreover, interfering with ZNF529-AS1 can significantly inhibit the invasion and migration of HCC cells. Next, to identify the downstream targets of ZNF529-AS1, we predicted that ZNF529-AS1 can regulate hsa-miR-561-5p by using the STARBASE database. hsa-miR-561-5p can be closely related to FBXO31 based on the relationship in the database, so we speculate that ZNF529-AS1 can regulate the expression of FBXO31. To verify the relationship between ZNF529-AS1 and FBXO31. We also verified the correlation between ZNF529-AS1 and FBXO31 by using the GEPIA database, and the results showed that ZNF529-AS1 was correlated with FBXO31 (*p* = 0.042). Therefore, we speculate that the expression of FBXO31 is correlated with ZNF529-AS1 and that FBXO31 is a downstream target of ZNF529-AS1 (Additional file 1: Fig. S4A–C). The expression of FBXO31 was downregulated after interfering with ZNF529-AS1 in HCC cells. ZNF529-AS1 may affect the invasion and migration of HCC cells by regulating FBXO31 expression. The regulatory mechanism of ZNF529-AS1 in HCC needs to be further explored to better understand its role in HCC.

## Summary

As a tumour-associated molecule, ZNF529-AS1 is highly expressed in HCC and is closely associated with the prognosis and immune infiltration of HCC. ZNF529-AS1 can affect the invasion and migration of HCC cells by regulating the expression of the downstream target FBXO31. ZNF529-AS1 may be a potential therapeutic target for HCC. The specific molecular mechanism of ZNF529-AS1 in HCC needs to be further studied.

## Supplementary Information

**Additional file 1.** The supplementary figure 1-figure 4.

**Additional file 2.** Supplementary table 1-table 2.

## Data Availability

Simulated datasets and the code used to produce the results presented in this paper are available at https://www.scidb.cn/s/VF7fmi.
