# Supplementary material for: LincRNA ZNF529-AS1 inhibits hepatocellular carcinoma via FBXO31 and predicts the prognosis of hepatocellular carcinoma patients
Source: BMC Bioinformatics. 2023 Feb 17;24:54. doi: 10.1186/s12859-023-05189-0 (PMC9938568; doi:10.1186/s12859-023-05189-0)
Supplement: Supplementary file 1 — Additional file 1. The supplementary figure 1-figure 4. [file 12859_2023_5189_MOESM1_ESM.docx]

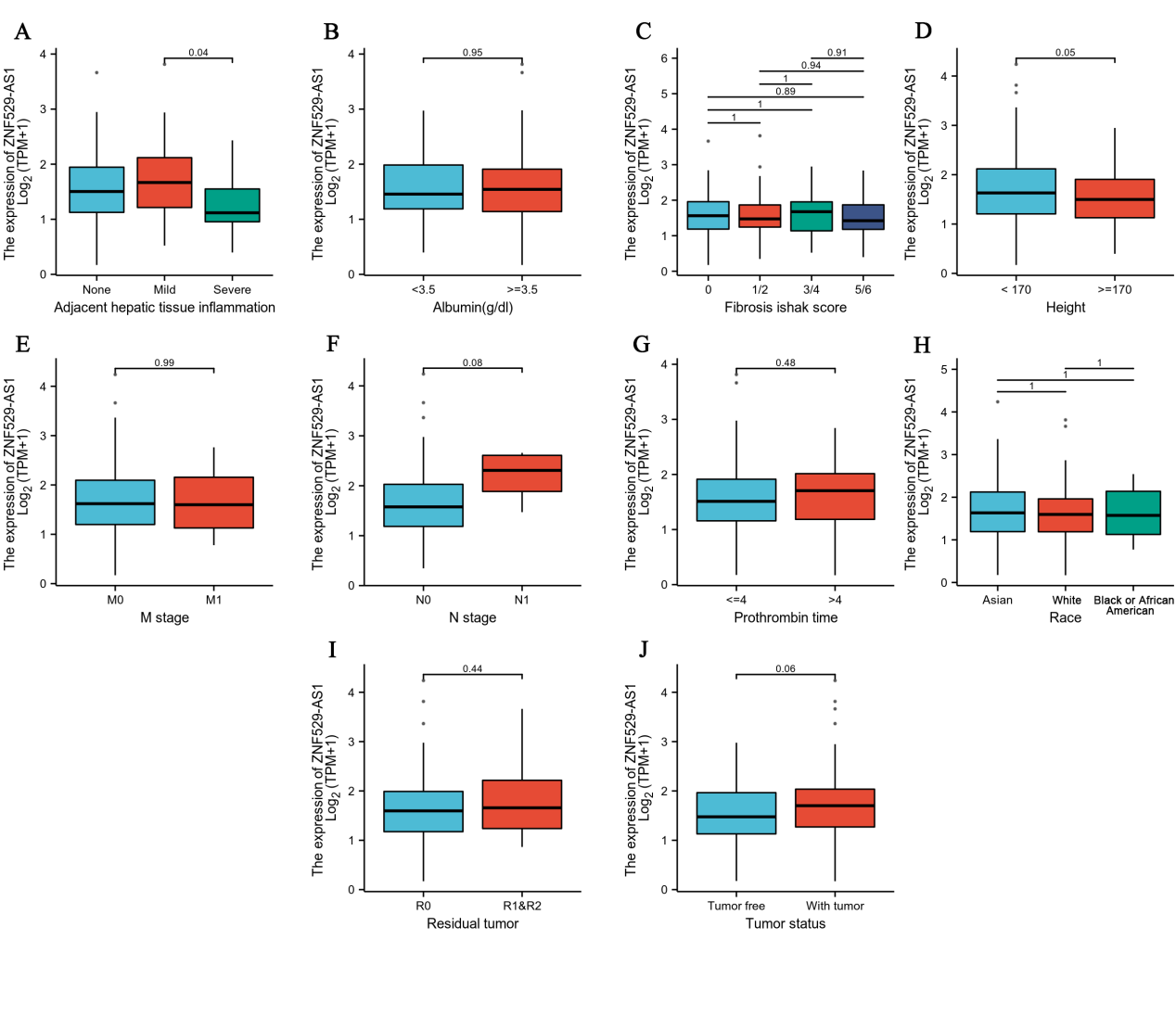


Supplementary Figure 1 Relationship between ZNF529-AS1 expression and clinical features, including (A) Adjacent hepatic tissue inflammation, (B) Albumin (g/dl), (C) Fibrosis ishak score, (D) Height, (E) M stage, (F) N stage, (G) Prothrombin time, (H) Race, (I) Residual tumor, (J) Tumor status.


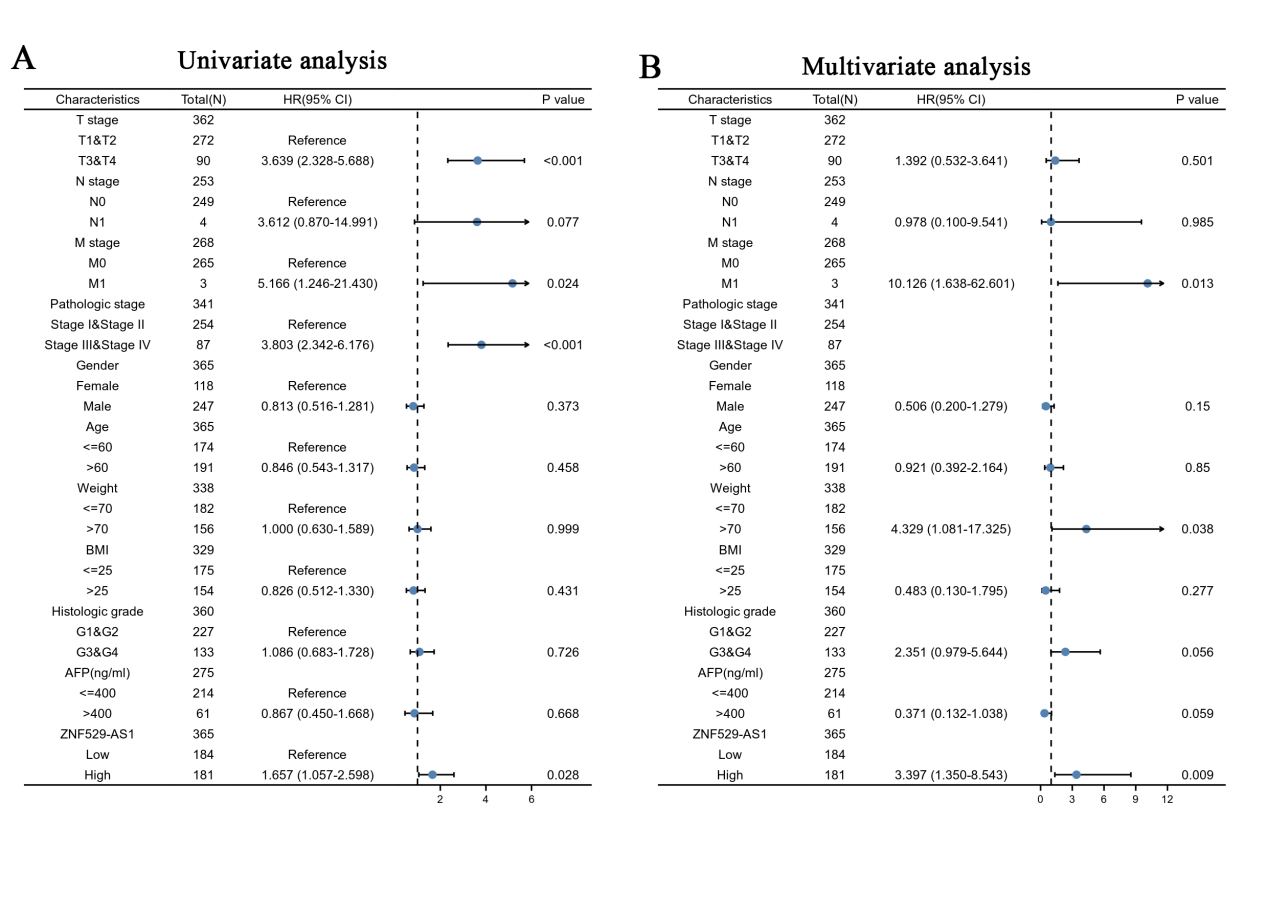


Supplementary Figure 2 ZNF529-AS1 can also be used as an independent prognostic factor for DSS in HCC patients. univariate (A) and multivariate (B) COX regression analysis of ZNF529-AS1 and different clinical characteristics.


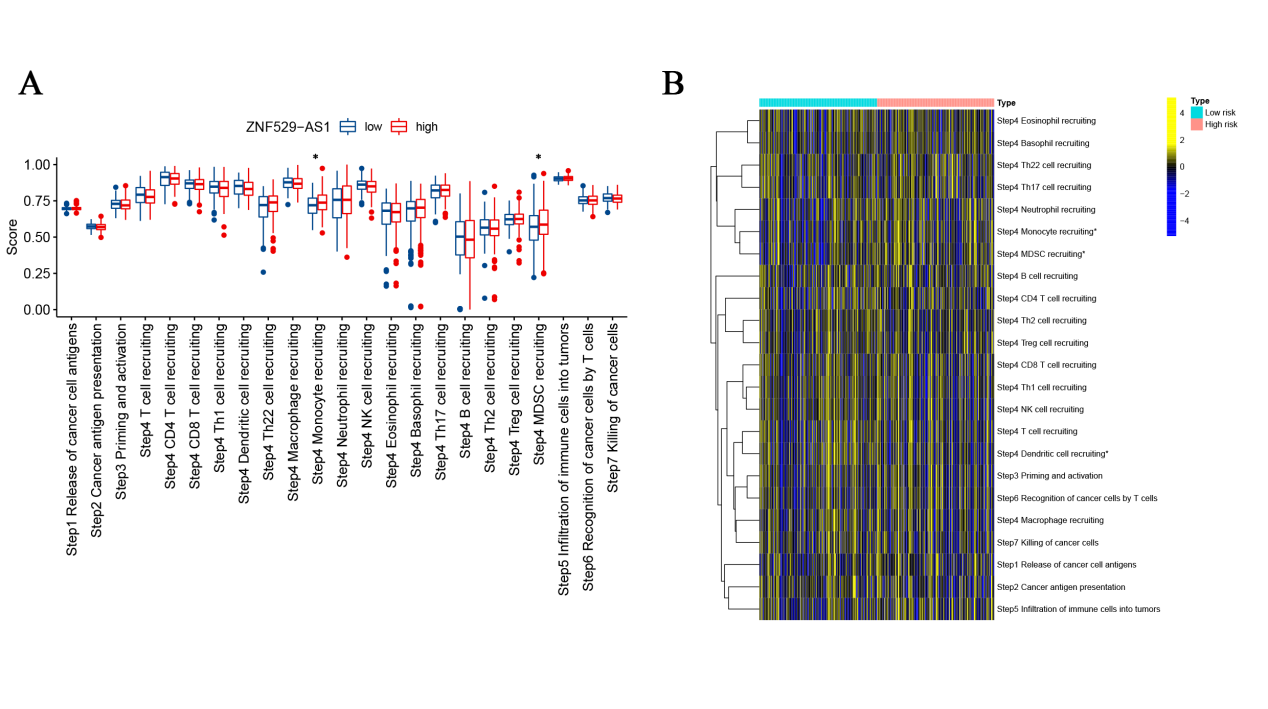


Supplementary Figure 3 Correlation between ZNF529-AS1 and various steps of the cancer immune cycle. (A) Analysis of the differences between the high and low ZNF529-AS1 expression groups at each step of the cancer immune cycle. (B) Correlation between the high and low expression groups in the cancer immune cycle.


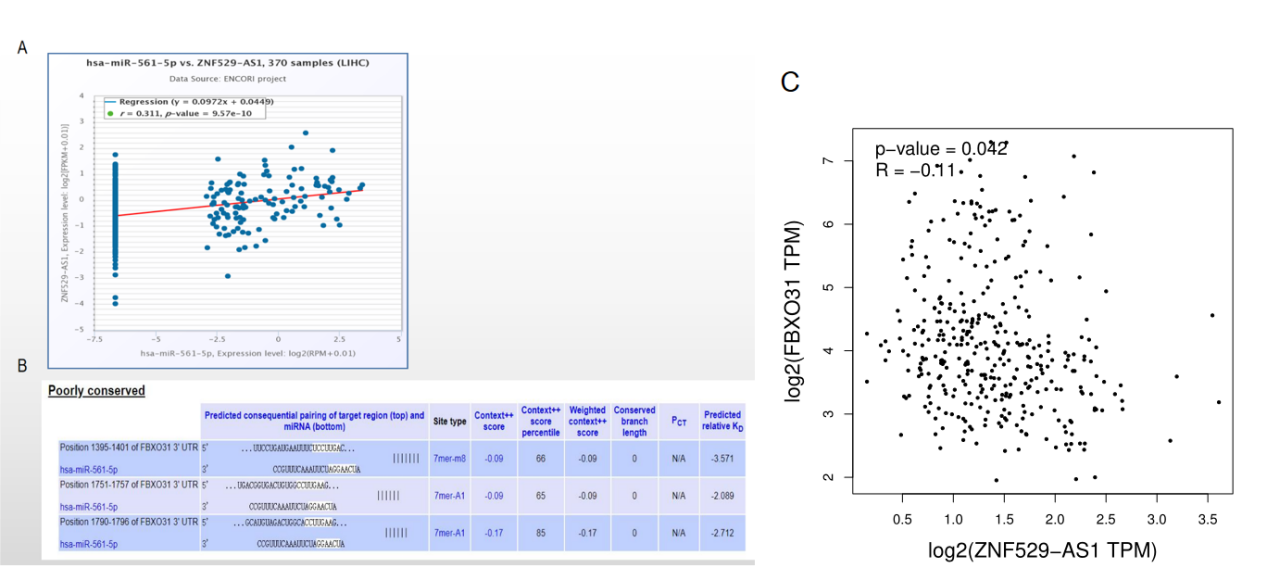


Supplementary Figure 4 Correlation between ZNF529-AS1 and FBXO31. (A, B) Correlation between ZNF529-AS1 and FBXO31 predicted by STARBASE database. (C) Correlation between ZNF529-AS1 and FBXO31 predicted by the GEPIA database
