# Supplementary material for: LincRNA ZNF529-AS1 inhibits hepatocellular carcinoma via FBXO31 and predicts the prognosis of hepatocellular carcinoma patients
Source: BMC Bioinformatics. 2023 Feb 17;24:54. doi: 10.1186/s12859-023-05189-0 (PMC9938568; doi:10.1186/s12859-023-05189-0)
Supplement: Supplementary file 2 — Additional file 2. Supplementary table 1-table 2. [file 12859_2023_5189_MOESM2_ESM.zip › Supplementary Table/Supplementary Table 1.docx]

Supplementary Table 1 Correlation analysis of ZNF529-AS1 expression with GO database

| Ontology | ID | Description | GeneRatio | BgRatio | pvalue | p.adjust | qvalue |
| --- | --- | --- | --- | --- | --- | --- | --- |
| BP | GO:0009410 | response to xenobiotic stimulus | 55/1451 | 292/18670 | 6.33e-10 | 3.34e-06 | 2.95e-06 |
| BP | GO:0042737 | drug catabolic process | 34/1451 | 140/18670 | 1.56e-09 | 4.12e-06 | 3.64e-06 |
| BP | GO:0023061 | signal release | 73/1451 | 462/18670 | 4.15e-09 | 7.31e-06 | 6.45e-06 |
| BP | GO:0046717 | acid secretion | 30/1451 | 124/18670 | 1.55e-08 | 1.43e-05 | 1.27e-05 |
| BP | GO:0010273 | detoxification of copper ion | 10/1451 | 15/18670 | 1.63e-08 | 1.43e-05 | 1.27e-05 |
| CC | GO:0045211 | postsynaptic membrane | 61/1532 | 323/19717 | 6.69e-11 | 2.19e-08 | 1.99e-08 |
| CC | GO:0097060 | synaptic membrane | 74/1532 | 432/19717 | 7.96e-11 | 2.19e-08 | 1.99e-08 |
| CC | GO:0098936 | intrinsic component of postsynaptic membrane | 28/1532 | 122/19717 | 1.54e-07 | 2.83e-05 | 2.57e-05 |
| CC | GO:0099055 | integral component of postsynaptic membrane | 27/1532 | 117/19717 | 2.27e-07 | 3.13e-05 | 2.83e-05 |
| CC | GO:0099699 | integral component of synaptic membrane | 31/1532 | 152/19717 | 5.68e-07 | 6.26e-05 | 5.67e-05 |
| MF | GO:0015267 | channel activity | 77/1391 | 456/17697 | 1.07e-10 | 5.49e-08 | 4.66e-08 |
| MF | GO:0022803 | passive transmembrane transporter activity | 77/1391 | 457/17697 | 1.19e-10 | 5.49e-08 | 4.66e-08 |
| MF | GO:0048018 | receptor ligand activity | 79/1391 | 482/17697 | 2.66e-10 | 8.18e-08 | 6.94e-08 |
| MF | GO:0022838 | substrate-specific channel activity | 72/1391 | 428/17697 | 5.18e-10 | 1.19e-07 | 1.01e-07 |
| MF | GO:0005216 | ion channel activity | 69/1391 | 416/17697 | 2.20e-09 | 4.06e-07 | 3.44e-07 |
