# Supplementary material for: LincRNA ZNF529-AS1 inhibits hepatocellular carcinoma via FBXO31 and predicts the prognosis of hepatocellular carcinoma patients
Source: BMC Bioinformatics. 2023 Feb 17;24:54. doi: 10.1186/s12859-023-05189-0 (PMC9938568; doi:10.1186/s12859-023-05189-0)
Supplement: Supplementary file 2 — Additional file 2. Supplementary table 1-table 2. [file 12859_2023_5189_MOESM2_ESM.zip › Supplementary Table/Supplementary Table 2.docx]

Supplementary Table 2 Correlation analysis of the expression of ZNF529-AS1 with the KEGG database

| Ontology | ID | Description | GeneRatio | BgRatio | pvalue | p.adjust | qvalue |
| --- | --- | --- | --- | --- | --- | --- | --- |
| KEGG | hsa04080 | Neuroactive ligand-receptor interaction | 67/643 | 341/8076 | 1.51e-12 | 4.51e-10 | 4.00e-10 |
| KEGG | hsa04976 | Bile secretion | 25/643 | 90/8076 | 1.73e-08 | 2.59e-06 | 2.29e-06 |
| KEGG | hsa00830 | Retinol metabolism | 21/643 | 68/8076 | 3.18e-08 | 3.17e-06 | 2.81e-06 |
| KEGG | hsa00140 | Steroid hormone biosynthesis | 17/643 | 61/8076 | 3.24e-06 | 2.42e-04 | 2.15e-04 |
| KEGG | hsa05204 | Chemical carcinogenesis | 20/643 | 82/8076 | 4.36e-06 | 2.61e-04 | 2.32e-04 |
| KEGG | hsa00980 | Metabolism of xenobiotics by cytochrome P450 | 19/643 | 77/8076 | 6.29e-06 | 3.14e-04 | 2.78e-04 |
| KEGG | hsa03320 | PPAR signaling pathway | 18/643 | 78/8076 | 2.95e-05 | 0.001 | 0.001 |
| KEGG | hsa04978 | Mineral absorption | 15/643 | 59/8076 | 4.06e-05 | 0.002 | 0.001 |
| KEGG | hsa00982 | Drug metabolism - cytochrome P450 | 15/643 | 71/8076 | 3.80e-04 | 0.013 | 0.011 |
| KEGG | hsa04724 | Glutamatergic synapse | 20/643 | 114/8076 | 5.99e-04 | 0.018 | 0.016 |
